# Kratom‐Associated Diffuse Alveolar Haemorrhage: A Clinical Image

**DOI:** 10.1002/rcr2.70439

**Published:** 2025-12-09

**Authors:** Venkatkiran Kanchustambham, Kara Johnson

**Affiliations:** ^1^ Sanford Health, Pulmonary & Critical Care Medicine Fargo North Dakota USA; ^2^ University of North Dakota Fargo North Dakota USA

**Keywords:** BAL, bronchoscopy, diffuse alveolar haemorrhage, Kratom, lung injury

## Abstract

A patient developed diffuse alveolar haemorrhage shortly after heavy kratom ingestion. Imaging and bronchoscopy confirmed haemorrhage. Vaping was excluded as a confounder.

## Graphical abstract

We report the case of a patient who developed diffuse alveolar haemorrhage shortly after heavy kratom ingestion. Imaging and bronchoscopy confirmed haemorrhage. Vaping was excluded as a confounder.

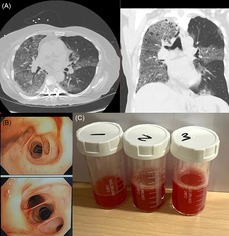

A middle‐aged woman presented with acute hypoxemic respiratory failure after ingesting large amounts of kratom daily. CT imaging demonstrated diffuse bilateral ground‐glass opacities. Bronchoscopy revealed hemorrhagic mucosa with progressively bloodier BAL aliquots, confirming diffuse alveolar haemorrhage (DAH). Both the patient and family verified she had not vaped for over 2 months prior to admission, making vaping‐related DAH unlikely. Workup for autoimmune, infectious, and vasculitic etiologies was negative. Heavy kratom ingestion remains the most plausible contributing factor (Figure 1) [1, 2].

**FIGURE 1:**
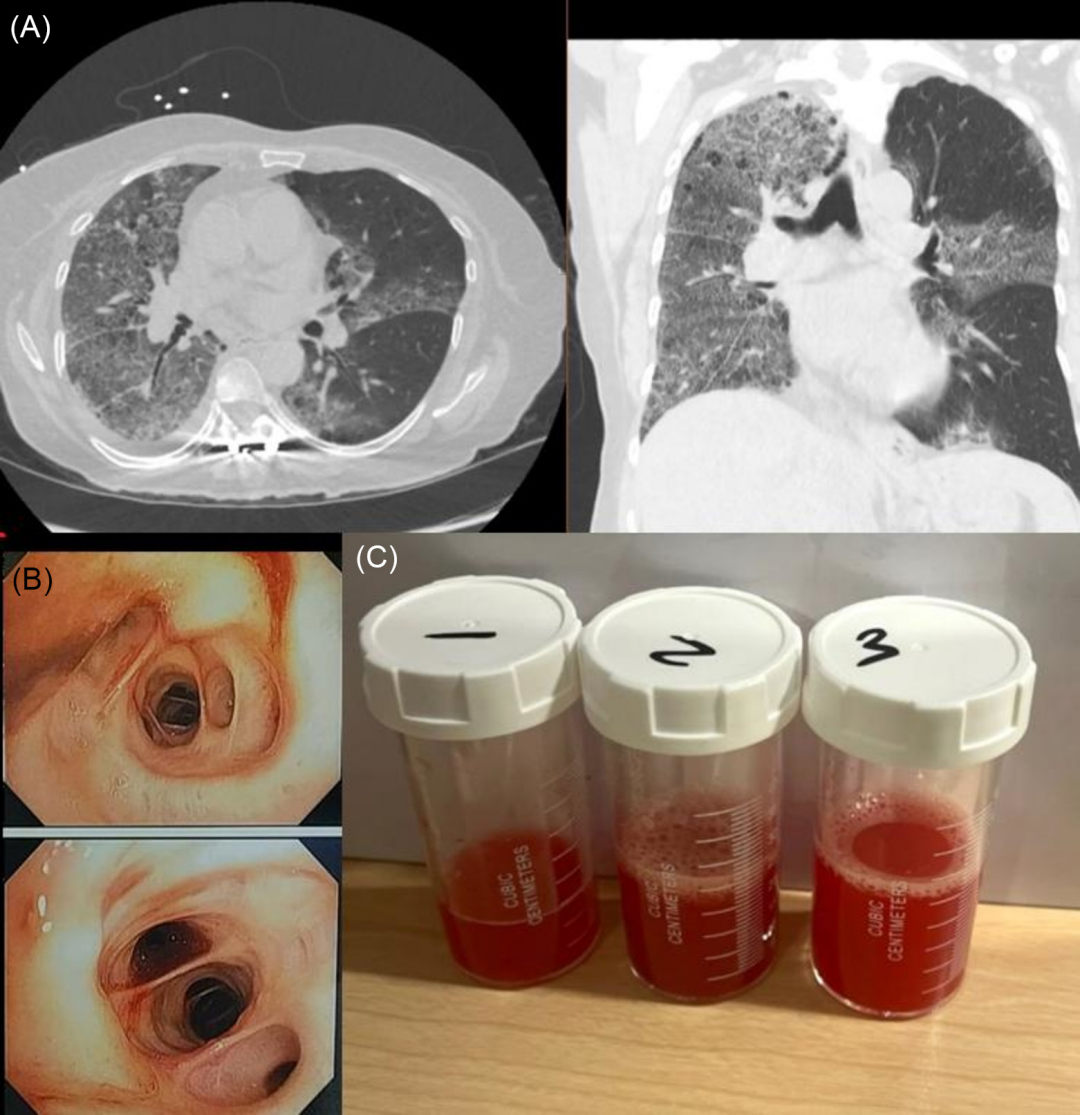
(A) Axial and coronal CT demonstrating diffuse bilateral ground‐glass opacities. (B) Bronchoscopy showing hemorrhagic mucosa. (C) BAL aliquots with progressively bloody appearance consistent with DAH.

## Author Contributions

**Venkatkiran Kanchustambham:** patient care, manuscript drafting. **Kara Johnson:** manuscript review. Both authors approved the final version.

## Consent

The authors declare that written informed consent was obtained for publication of this manuscript and images using the journal‐required consent form.

## Conflicts of Interest

The authors declare no conflicts of interest.

## Data Availability

The data that support the findings of this study are openly available in Google Scholar at https://scholar.google.com/citations?user=JGwxTD0AAAAJ&hl=en&oi=ao.
